# Absence of right common iliac vein causing suprapubic and scrotal varices in a young athletic man

**DOI:** 10.1016/j.jvscit.2021.09.003

**Published:** 2021-10-02

**Authors:** Saad Balamane, Peter Brown, David Zelt, Michael Yacob

**Affiliations:** a Faculty of Health Sciences, Queen's University, Kingston, Ontario, Canada; b Division of Cardiovascular Surgery, Queen's University, Kingston Health Sciences Centre, Kingston, Ontario, Canada

**Keywords:** Right common iliac vein, Varicosities

## Abstract

We have described the case of a 26-year-old man who had presented to his primary care physician with persistent, painful varices across his lower abdomen and bilateral tender scrotal varicoceles, which intensified with exercise. Thorough investigations revealed a congenitally atretic right common iliac vein with right-to-left collateralization of the femoral and internal iliac veins. This shunting resulted in the development of suprapubic and pelvic and gonadal varicosities, which provided a critical venous outflow pathway for his right lower extremity. Heightened vigilance is, hence, paramount if our patient requires future abdominal and urologic procedures. Moreover, the present case has highlighted the importance of considering deep system venous anomalies when determining the differential diagnosis for venous diseases.

The global incidence of congenital vascular malformation is 1.5%, although geographic variations exist.^1^ A congenital vascular malformation is an inborn error of vascular morphogenesis, ensuing in true structural anomalies. Although arterial and venous malformations are possible, congenital venous malformations account for two thirds of worldwide cases. Specifically, the congenital absence of the right common iliac vein is a distinctly rare clinical entity, with only a few isolated cases reported in the literature, most of which were discovered during cadaveric dissection.^2^^,^^3^ Thrombosis or congenital anomalies of the common iliac vein can present as suprapubic varicosities and should prompt further clinical investigation. We have described the case of a congenitally absent right common iliac vein, with persistent right external and iliac veins. As a result, our patient developed a unique collateralization in the pelvic and suprapubic venous systems, resulting in both scrotal and subcutaneous suprapubic varices. The patient provided written informed consent for the report of his case details and imaging studies.

## Case report

**Fig 1:**
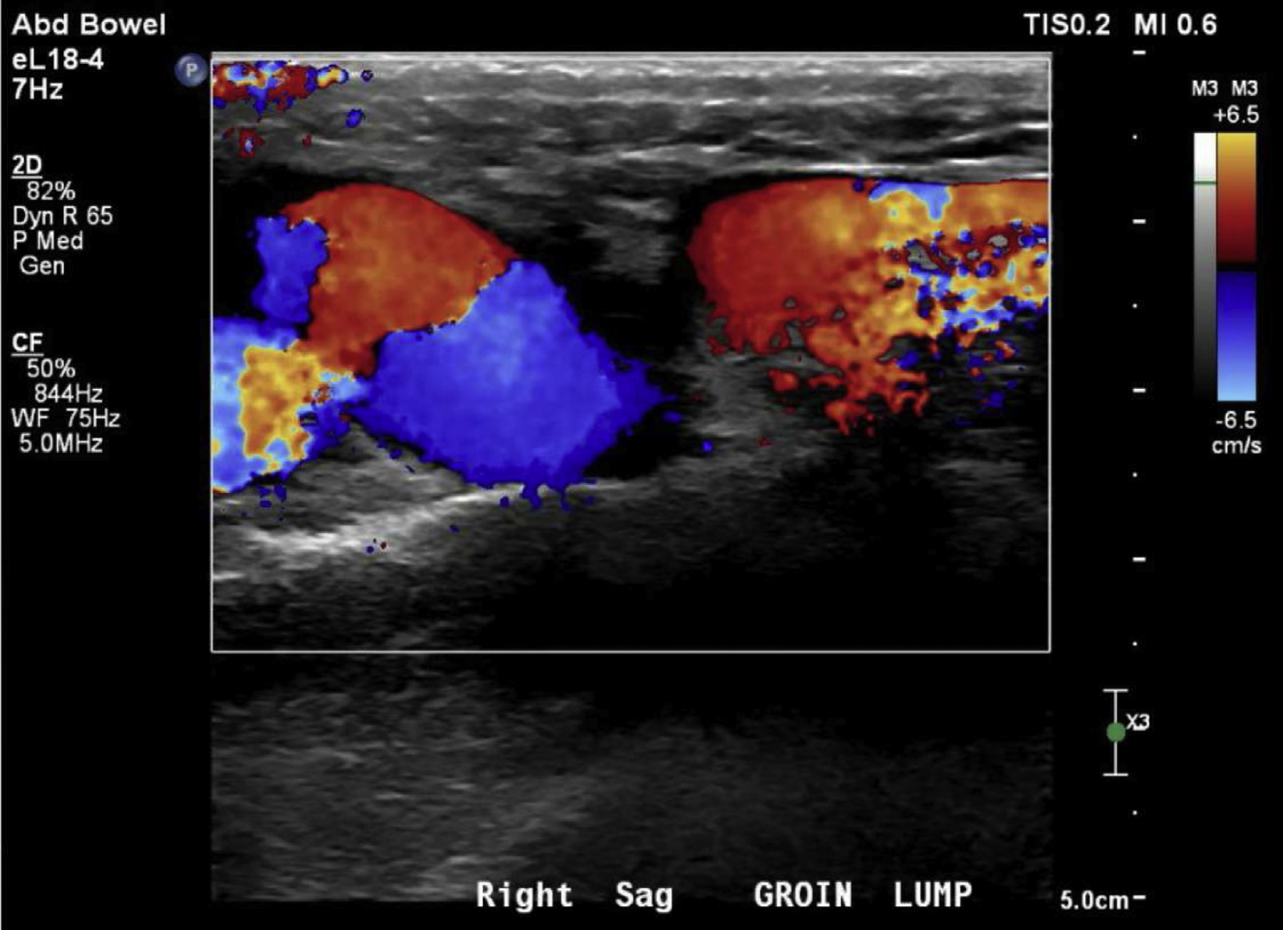
Color duplex ultrasound image of right saphenous femoral junction showing large, varicose femorofemoral collateral with right to left shunting of venous blood.

**Fig 2:**
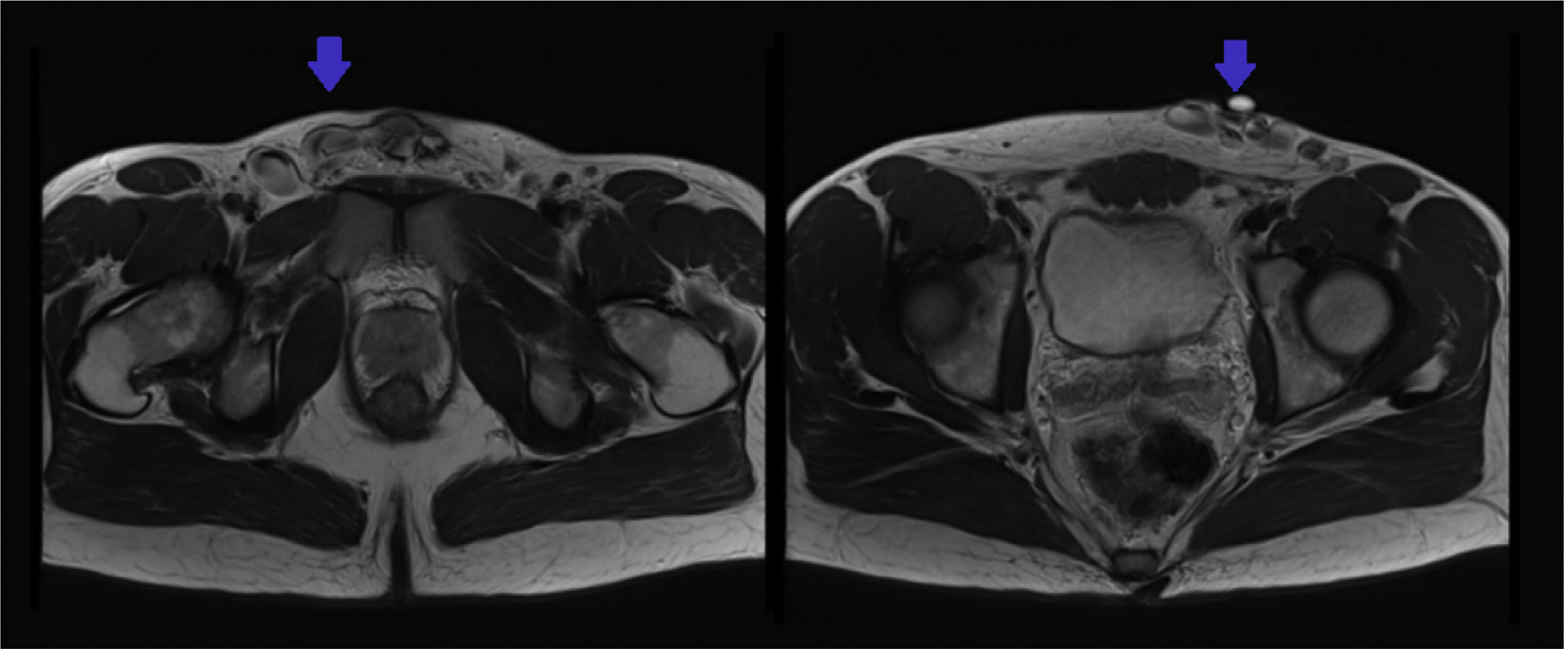
T2-weighted magnetic resonance venogram (MRV) of pelvis showing elaborate suprapubic collateral vessels (*arrow*) in both still frames in an axial projection.

The patient, an otherwise healthy 26-year-old man, had presented to his family physician with persistent, uncomfortable varices along his lower abdomen and bilateral scrotum. He described the symptoms as heaviness, swelling, and burning in these regions. These symptoms were exacerbated by large bowel movements or exercise and persisted for ∼30 minutes after a heavy cardiovascular routine. He had had these symptoms for his entire life; however, they had worsened when he increased his athletic extracurricular activities at the age of 13 years. On physical examination, he was a slender man of athletic build. He had pronounced varicosities in both groins and over his pubic tubercle. The scrotal and penile examination demonstrated pronounced engorgement of his testicular veins within the scrotum. His lower extremities did not demonstrate any evidence of venous insufficiency. The arterial examination findings were unremarkable. The abdominal examination did not demonstrate findings concerning for central abdominal masses. The primary care physician initially suspected that his inguinal enlargement was possibly an abdominal hernia. This tentative diagnosis was explored using duplex ultrasound, which excluded the presence of an inguinal or femoral hernia. However, the duplex ultrasound findings revealed enlarged superficial veins along the length of the suprapubic abdominal region (Fig 1). These findings prompted magnetic resonance venography (MRV) with gadolinium contrast of the pelvis to evaluate retroperitoneal causes of atypical venous anatomy. MRV displayed tortuous dilated structures within the subcutaneous fat of the inferior ventral abdominal wall above the pubic symphysis compatible with venous varices (Fig 2). These structures communicated bilaterally with the common femoral veins through large tributaries in the saphenofemoral junctions. Some of the findings were in keeping with common iliac vein occlusion; however, the findings were ultimately indeterminant. No other retroperitoneal anomalies were identified. Based on these findings, his family physician referred him to vascular surgery for further evaluation and consideration of treatment of his symptomatic varices.

**Fig 3:**
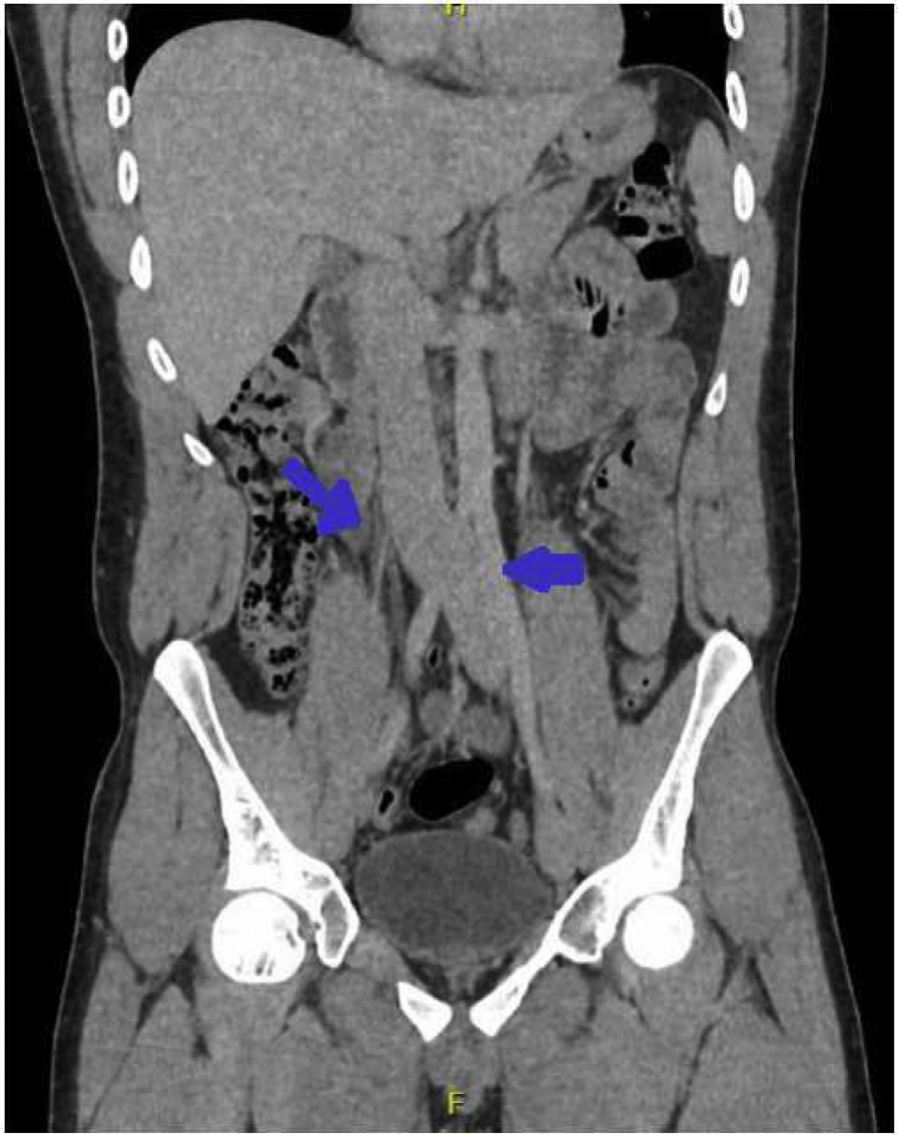
Coronal projection of computed tomography venogram (CTV) of abdomen and pelvis demonstrating absence of the right common iliac vein and a slightly dilated left common iliac vein.

Because of the strong clinical suspicion of a central cause of occlusion, outpatient computed tomography venogram (CTV) of the abdomen and pelvis was ordered. The CTV showed the absence of the right common iliac vein. Although small in caliber, the right internal and external iliac veins were present. Prominent internal iliac vein collateral vessels (right to the left) were present in the presacral space, which caused varices to develop in the pelvic, scrotal, and penile venous network. The CTV further revealed that the venous collateral vessels in the lower abdominal wall overlying the pubic symphysis had originated from the right saphenofemoral junction, draining into the left saphenofemoral junction (Fig 3). The left internal iliac and external iliac veins were present and slightly engorged. The left common iliac vein and inferior vena cava were patent, without unusual features. No evidence was found of other relevant abdominal pathology or anomaly.

The patient was educated regarding his aberrant anatomy and instructed to forbid ligation or injection of his suprapubic varices, given this important collateral network. He was also instructed to notify surgeons planning future pelvic or urologic surgery of his large pelvic venous collateral vessels. For symptom relief, we recommended a pantyhose height, 20- to 30-mm Hg graduated compression garment. Should the patient progress to debilitating chronic venous insufficiency, suprapubic skin necrosis, or infertility, we are prepared to proceed with iliocaval bypass. Nonetheless, this would be a significant endeavor and the current clinical findings do not warrant major surgery. No clinical follow-up was arranged; however, the patient was given our office contact information and educated regarding the signs and symptoms that would warrant consideration of surgical intervention.

## Discussion

Any malformation of the cardinal veins can lead to agenesis of the common iliac veins.^2^ Specifically, anomalies of the iliac confluence of the posterior cardinal vein can result in the congenital absence of the right common iliac vein, as was seen in our patient.^2^^,^^3^

Although previous studies have described the congenital absence of the common iliac veins and their branches, the anatomy of our patient was unique in several aspects.^2^^,^^3^ He had patent right external and internal iliac veins. Thus, in addition to the right-to-left femorofemoral collateral vessels, he had developed right-to-left internal ilioiliac vein collaterals through his pelvic venous system. As such, he had scrotal varices and suprapubic varicosities. A previously reported pediatric case by Yahyayev et al^3^ showed that their patient had congenital absence of the right external and internal iliac veins, in addition to his right common iliac vein. However, in their case, the patient's external genitalia did not have varicosities. Similar to that patient, our patient has an increased risk of iatrogenic injury during abdominal and pelvic surgical procedures, given the presence of pelvic and lower abdominal wall varicosities.^4^ The scrotal varicosities also result in increased risk during urologic procedures.^5^

The findings of varicosities over the pubic tubercle, with the prominence of superficial veins with sparing of the lower extremities, should raise concerns for central venous occlusion (CVO). In patients without a history of central venous catheterization, a CVO might indicate the presence of extrinsic compression of the venous system, such as lymphoma.^6^ Another uncommon cause of CVO is May-Thurner syndrome, which traditionally compresses the left common iliac vein by the right common iliac artery. However, in some cases, the arterial compression can cause right common iliac vein compression.^7^ Therefore, it is of paramount importance to rule out the presence of a CVO in patients with suspected common iliac vein thrombosis.^6^ The incidence of iliofemoral DVT is far greater in patients with vascular structural anomalies; hence, the distinction between vein compression syndrome and agenesis or atresia of the iliofemoral vessels is important.^6^^,^^8^ With CVO and subsequent DVT, these patients will be at greater risk of developing post-thrombotic syndrome and chronic venous insufficiency.^9^

In patients without a CVO but with congenital venous malformations, such as in our patient, surgical intervention remains the most pressing life-threatening risk.^2^, ^3^, ^4^^,^^8^ Magnetic resonance imaging modalities, such as MRV, are radiation-free and highly valuable for appreciating the patient's anatomy and ruling out DVT and abdominopelvic masses.^3^ The CTV is also suitable, as noted in the present case. Finally, conventional venography remains the reference standard for imaging to investigate atypical venous outflow tracts because it can more accurately demonstrate all collateral pathways that would otherwise be too difficult to discern using either MRV or CTV. In addition to gathering diagnostic information, we could consider performing embolization of the gonadal veins to treat the varicoceles. The benefit of this procedure would be to provide significant symptomatic relief and, in the rare case of infertility, could potential help restore reproductive function. Gonadal embolization in such cases is not completely benign. If an adequately developed collateral network is not present, a serious risk exists of fulminant venous thrombosis, which could require urgent orchiectomy.

Surgeons performing pelvic, urologic, and abdominal surgery should proceed with caution to prevent considerable hemorrhage from iatrogenic injury to the collateral veins.^4^^,^^5^^,^^8^

## Conclusions

The findings from the present case have reinforced that knowledge of venous variants is imperative to avoid iatrogenic injury to the venous system. Although the symptoms reported by our patient were relatively benign, the proximity of such critical venous collateral vessels to the abdominal wall and scrotum should prompt consulting surgeons to demonstrate increased prudence.
